# The Role of HBZ in HTLV-1-Induced Oncogenesis

**DOI:** 10.3390/v8020034

**Published:** 2016-02-02

**Authors:** Tiejun Zhao

**Affiliations:** 1 College of Chemistry and Life Sciences, Zhejiang Normal University, 688 Yingbin Road, Jinhua 321004, China; tjzhao@zjnu.cn; Tel.: +86-579-8229-1410; 2 Key Lab of Wildlife Biotechnology and Conservation and Utilization of Zhejiang Province, Zhejiang Normal University, 688 Yingbin Road, Jinhua 321004, China

**Keywords:** HBZ, HTLV-1, ATL

## Abstract

Human T-cell leukemia virus type 1 (HTLV-1) causes adult T-cell leukemia (ATL) and chronic inflammatory diseases. HTLV-1 bZIP factor (HBZ) is transcribed as an antisense transcript of the HTLV-1 provirus. Among the HTLV-1-encoded viral genes, HBZ is the only gene that is constitutively expressed in all ATL cases. Recent studies have demonstrated that HBZ plays an essential role in oncogenesis by regulating viral transcription and modulating multiple host factors, as well as cellular signaling pathways, that contribute to the development and continued growth of cancer. In this article, I summarize the current knowledge of the oncogenic function of HBZ in cell proliferation, apoptosis, T-cell differentiation, immune escape, and HTLV-1 pathogenesis.

## 1. Introduction

**Figure 1:**
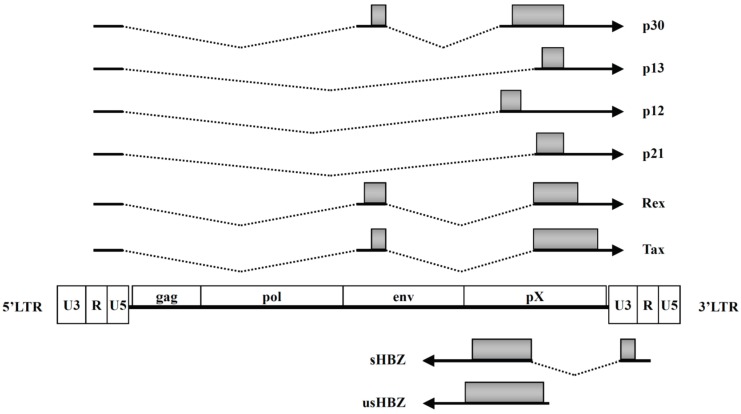
Regulatory and accessory genes encoded by HTLV-1. p12, p13, p30, Rex, p21, and Tax are transcribed from the 5' long terminal repeat (LTR). HBZ is located on the complementary proviral strand and transcribed from the 3' LTR. Spliced (s) and unspliced (us) HBZ are shown. Shaded boxes represent their coding regions.

Human T-cell leukemia virus type 1 (HTLV-1) is the first identified pathogenic retrovirus that is etiologically associated with two major diseases: adult T-cell leukemia (ATL) and a progressive myelopathy called HTLV-1-associated myelopathy/tropical spastic paraparesis (HAM/TSP) [1,2,3,4]. Four types of HTLVs, named HTLV-1, HTLV-2, HTLV-3, and HTLV-4, have been characterized [5]. However, their oncogenic properties are different. The HTLV-1 genome, in addition to the structural genes *gag*, *pol*, and *env*, carries a region at its 3' end, which is designated the pX region, and encodes several accessory genes, including *tax*, *rex*, *p12*, *p21*, *p30*, *p13*, and *HTLV-1bZIP factor* (*HBZ*) (Figure 1) [6,7]. In the 35 years since the discovery of HTLV-1, researchers have mainly focused on Tax, a *trans*-acting viral regulatory protein, due to its pleiotropic functions in viral replication and cellular transformation [8,9,10]. However, approximately 60% of ATL cases lack Tax expression because of genetic and epigenetic changes in the proviral genome of HTLV-1, suggesting that Tax is not essential for the maintenance of ATL [5,11]. In 2002, a new open reading frame (ORF) was identified on the minus strand of HTLV-1, corresponding to a region complementary to Tax, which encodes a novel basic leucine zipper factor named HBZ [6]. Studies have shown that HBZ is consistently expressed in all ATL cases [12]. In some of the ATL cases, HTLV-1 genes, except HBZ, are mutated by APOBEC3G [13]. Moreover, Tax is an immunodominant antigen of this virus and is the major target of cytotoxic T lymphocytes (CTLs) [14]. Thus, Tax expression is frequently silenced during the long clinically latent period of the virus [5,11]. In contrast, the immunogenicity of HBZ protein is low compared with that of Tax protein [15,16,17]. The constitutive expression of HBZ, by way of suppressing major HTLV-1 sense genes including Tax, could help the virus escape from the host’s immune surveillance and thus promote spread of infection [5,18,19]. These observations suggest that the HBZ gene is essential for cellular transformation and leukemogenesis of HTLV-1. In this review, I will highlight recent advances in our understanding of how HBZ contributes to HTLV-1 oncogenesis.

## 2. Characteristics of HBZ

### 2.1. Expression of HBZ in ATL

In order to achieve successful transformation, HTLV-1 uses its genome very efficiently to encode multiple viral genes. Tax, an HTLV-1 plus strand-encoded viral gene, has been implicated in the leukemogenesis of HTLV-1 as it has growth-promoting activities and the ability to immortalize various types of cells *in vivo* [5,8,9,20]. However, Tax is the major target of CTLs [14]. Thus, ATL cells frequently lose Tax expression by several mechanisms. First, the 5' LTR of HTLV-1 provirus is reported to be deleted in 39% of ATL cases, resulting in the loss of Tax in ATL [21,22]. The second mechanism involves the nonsense mutation, deletion, and insertion of the *tax* gene in ATL cells [11,23]. The third mechanism includes DNA hypermethylation and histone modification of the 5' LTR of HTLV-1, which silences viral gene transcription [24,25]. Therefore, Tax may not be necessary for the development of ATL. On the contrary, the 3' LTR is conserved and unmethylated in all ATL cases [13,26]. Previous study demonstrated that the HBZ gene was expressed in all ATL cells and that HBZ gene knockdown inhibited the proliferation of HTLV-1-infected cells, indicating that HBZ may play a critical role in HTLV-1-mediated oncogenesis [12]. Sp1 binding sites in the 3' LTR of HTLV-1 have been demonstrated to be critical for HBZ promoter activity [26]. As Sp1 is a well-known regulator of housekeeping genes, the transcription of the HBZ gene may be relatively constant.

The expression of HBZ has been correlated with HTLV-1 proviral load [27,28]. Kinetic analyses of the HBZ transcript in HTLV-1-infected rabbits revealed that HBZ was detected at relatively low levels early after infection but gradually increased and stabilized, whereas other viral genes were maintained continuously at a low level [29]. Moreover, there is a correlation between the level of HBZ expression and the severity of HAM/TSP, indicating that high levels of HBZ may be associated with a greater risk of HAM/TSP [27].

A 5' RACE experiment identified two different HBZ transcripts in ATL; one is spliced (sHBZ) and the other is unspliced (usHBZ) (Figure 1) [12,30,31]. The s*HBZ* gene transcript level was approximately four times higher than the us*HBZ* gene transcript [28]. Consistently, the level of the usHBZ protein was much lower than that of sHBZ [28]. Furthermore, Western blot analyses could detect only the sHBZ protein in ATL cell lines [32,33].

### 2.2. Structure of HBZ

HBZ was first identified as a binding partner for the cAMP-response element binding protein-2 (CREB-2) by yeast two-hybrid screening [6]. Promoters for both the s*HBZ* and us*HBZ* genes are TATA-less [26]. The transcription factor Sp1 has been demonstrated to be important for TATA-less promoter activity [34,35]. Consistent with these observations, the sHBZ promoter is activated by the constitutively expressed Sp1 protein [26]. Moreover, Tax can activate the activity of sHBZ and usHBZ gene promoters through Tax-responsible elements (TREs) in the U3 region of HTLV-1 3' LTR [26,36]. However, Tax-mediated activation of antisense transcription is weaker than its activation of HTLV-1 sense transcription [26]. In addition, Tax-induced HBZ expression is influenced by the integration site in the host genome [36].

The sHBZ transcript is translated into a polypeptide of 206 amino acids, and the protein product of usHBZ is 209 amino acids long. Both HBZ isoforms contain three domains: activation domain (AD), central domain (CD), and basic leucine zipper domain (bZIP) [6,37]. The LXXLL-like motif, which is located at the N-terminal activation domain, is critical for HBZ-mediated activation of Smad3/TGF-β pathway through binding to CBP/p300 [38]. There are three nuclear localization signals (NLSs) that are responsible for the nuclear localization of HBZ protein: two regions in the CD domain and a basic region in the bZIP domain [37]. A recent study demonstrated that HBZ contains a functional nuclear export signal (NES) sequence within its N-terminal region and disrupts the cellular autophagic response in the cytoplasm [39]. The sHBZ and unHBZ proteins differ in only seven amino acids at their N-terminal AD domains. The expression level of sHBZ is four times higher than that of usHBZ in ATL cells [28]. It is well known that different post-translational N-terminal modifications of proteins affect their half-lives. The half-life of sHBZ is much longer than that of usHBZ [26]. Thus, the N-terminal AD domain differences may be responsible for the distinct protein levels observed. In addition, Dissinger *et al.* [40] used an affinity-tagged protein and mass spectrometry method to identify seven modifications of HBZ protein. However, none of the identified post-translational modifications affected HBZ stability or its regulation of signaling pathways.

## 3. Oncogenic Properties of HBZ

### 3.1. Suppression of Viral Transcription

**Figure 2:**
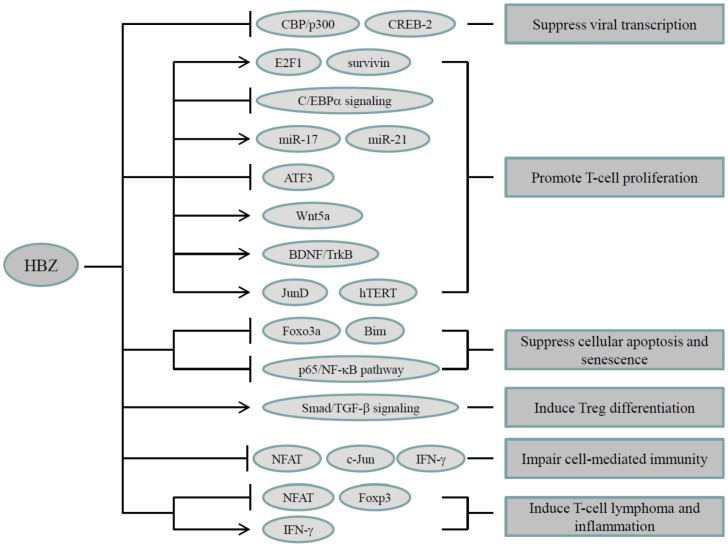
Oncogenic function of HBZ. HBZ fulfills its oncogenic functions mainly through regulating HTLV-1 5' LTR transcription and modulating a variety of cellular signaling pathways that are related to cell growth, apoptosis, immune escape, T-cell differentiation, and HTLV-1 pathogenesis. Detailed descriptions can be found in the text.

CREB-2 (ATF-4) was identified as a binding protein to HBZ [6]. Dimerization between HBZ and CREB-2 prevented CREB-2 from binding to a Tax-responsive element (TxRE) site in the HTLV-1 5' LTR, resulting in the suppression of Tax-mediated HTLV-1 5' LTR activation (Figure 2) [6]. Moreover, HBZ also represses CREB transcription from a cellular cyclic AMP-responsive element (CRE) in the cyclin D1 promoter, extending the inhibitory function of HBZ to CREB-dependent transcription of cellular genes [41]. The bZIP domain of HBZ contributes to this repression. In addition, Clerc *et al.* reported that HBZ interacts with p300/CBP and disrupts the interaction between Tax and p300/CBP, thereby inhibiting the Tax-dependent viral transcription [42]. Two LXXLL-like motifs located within the NH2-terminal region of HBZ mediate the interaction specifically through the KIX domain of the p300/CBP coactivator [42]. The two LXXLL motifs in the AD domain of HBZ promote binding to the mixed-lineage leukemia (MLL) surface of the KIX domain [43]. Formation of this interaction inhibits binding of MLL to the KIX domain while enhancing the binding of the transcription factor c-Myb to the opposite surface of KIX [43].

### 3.2. Promotion of T-Cell Proliferation

The development and continued growth of cancers involves altered rates of cell proliferation [44,45]. Multiple studies have demonstrated that HBZ plays critical roles in ATL leukemogenesis through pleiotropic actions, which include the promotion of cell proliferation [18,19]. Satou *et al.* reported that the HBZ gene enhances proliferation of T cells *in vitro* and *in vivo* [12]. Stable expression of HBZ gene increases Kit 225 cell proliferative capacity. Furthermore, repression of HBZ expression in ATL cell lines by shRNA inhibited the growth of ATL cells. Mutant analyses showed that HBZ promoted proliferation of T cells as a messenger RNA [12]. Growth-promoting activity was observed only in the cells expressing sHBZ and not in usHBZ-expressing cells [26]. Moreover, the percentage of CD4^+^ T lymphocytes increased in splenocytes of HBZ transgenic (HBZ-Tg) mice, and HBZ-expressing T-cells proliferated more rapidly than those of non-transgenic mice [12,46]. In NOD/SCID^γ^^c-/-^ (NOG) mice, HBZ-expressing SLB-1 cells engrafted to form solid tumor masses, but tumor formation was reduced significantly in animals challenged with HBZ-knockdown SLB-1 cells [32]. Thus, these data collectively indicate that HBZ expression enhances the proliferative capacity of HTLV-1-infected cells and plays a critical role in cell survival.

Recently, Mitobe *et al.* reported that *HBZ* RNA increased the number of CD4^+^ T cells and attenuated cell death by activating the transcription of the *survivin* gene, which inhibits apoptosis (Figure 2). Moreover, the first 50 base pairs of the *HBZ* coding sequence are required for RNA-mediated cell proliferation [47].

Accumulating evidence suggests that the HBZ protein modulates cell growth through forming heterodimers with several host factors, such as C/EBPα and ATF3 (Figure 2) [48,49]. C/EBPα has emerged as an important negative regulator of cell proliferation in different cancers. In ATL cells, HBZ overcame the suppressive effect of C/EBPα on cell growth, leading to cell proliferation. A suggested underlying mechanism is that HBZ inhibits C/EBPα signaling activation by interacting with C/EBPα and diminishes its DNA binding capacity [48]. ATF3, an HBZ-binding protein, is constitutively expressed in ATL cell lines and fresh ATL cases. HBZ attenuates the negative effects of ATF3, allowing ATF3 to promote the proliferation of ATL cells by mechanisms that upregulate the expression of genes that are critical for mediating the cell cycle and cell death [49].

Increasing evidence has demonstrated that microRNAs (miRNA) play critical roles in the development of cancer [50]. In ATL, expression profiling of microRNA revealed that oncogenic miRNAs, including miR-17 and miR-21, are overexpressed in HTLV-1-infected T cells. These two miRNAs are post-transcriptionally upregulated by HBZ, and HBZ/miRNA-mediated downregulation of OBFC2A expression triggers both cell proliferation and genomic instability (Figure 2) [51].

Polakowski *et al.* reported that HBZ activates expression of neurotrophin BDNF. Moreover, HBZ promotes a BDNF/TrkB autocrine/paracrine signaling loop in HTLV-1-infected T cells, leading to the survival of these cells (Figure 2) [52].

In addition, HBZ suppresses the canonical Wnt pathway while enhancing the proliferation and migration of ATL cells by increasing expression of the noncanonical Wnt5a. These observations suggest that perturbation of the Wnt signaling pathways by HBZ is associated with the leukemogenesis of ATL (Figure 2) [53].

HBZ suppresses AP-1 signaling pathway, which is mediated by c-Jun and JunB [54,55,56]. However, HBZ can activate JunD-induced transcription by forming heterodimers with JunD, resulting in the activation of JunD-dependent cellular genes including human telomerase reverse transcriptase (hTERT) (Figure 2) [57,58,59]. The activation of telomerase by HBZ may contribute to the maintenance of leukemic cells.

### 3.3. Suppression of Cellular Apoptosis and Senescence

Defective apoptosis represents a major causative factor in the development and progression of cancer [44,45]. HTLV-1 suppresses apoptosis of infected cells by the interactions of viral proteins with host factors [60]. HBZ has been demonstrated to inhibit the transcription of a proapoptotic gene Bim, resulting in the decreased activation-induced cellular apoptosis. By interacting with FoxO3a, which is a transcriptional activator of the Bim gene, HBZ attenuates the DNA binding ability of FoxO3a and sequesters the inactive form of FoxO3a in the nucleus. Further study has identified that HBZ inhibited the expression of Bim by epigenetic alterations and histone modifications in the Bim promoter region. Thus, it may be advantageous for HBZ to suppress the apoptosis of ATL cells (Figure 2) [61].

Tax-induced nuclear factor-κB (NF-κB) activation plays a central role in HTLV-1-mediated transformation of human T cells [62,63]. However, hyper-activation of NF-κB by Tax triggers a defense mechanism that induces cellular senescence [64]. By contrast, HBZ delays or prevents the onset of Tax-induced cellular senescence by down-regulating NF-κB signaling (Figure 2). Zhao *et al.* reported that HBZ protein selectively inhibits Tax-mediated classical NF-κB activation by inhibiting p65 DNA binding capacity and by promoting expression of the PDLIM2 E3 ubiquitin ligase, which results in p65 degradation [65]. Inhibition of p65 acetylation by HBZ also contributes to the repression of the classic NF-κB pathway [66]. A recent study demonstrated that HBZ maintains viral latency by down-regulating Tax-mediated NF-κB activation and by inhibiting Rex-induced expression of viral proteins [67]. Taken together, these observations suggest that HBZ modulates Tax-mediated viral replication and NF-κB activation, thus allowing HTLV-1-infected cells to proliferate and persist.

In ATL, HBZ-mediated suppression of the classical NF-κB pathway decreases the expression of some genes associated with innate immunity and inflammatory responses [65]. NF-κB signaling is a well-established mediator of host immunity [68]. Therefore, HTLV-1 may facilitate escape from the host immune attack by suppressing the classical NF-κB pathway in such a manner.

Current data support the view that Tax may facilitate cell proliferation and survival in the early stage of HTLV-1 infection through activating NF-κB pathway. Nevertheless, Tax protein is the main target of the host’s CTLs. Therefore, it is plausible that in the late stages of leukemogenesis, ATL cells that lack Tax expression are selected to emerge. In these cells, NF-κB-inducing kinase (NIK), a known activator of NF-κB, may replace Tax to maintain the constitutive activation of NF-κB, a hallmark of leukemic ATL cells [69]. At this stage, the mitogenic activity of HBZ may be required to maintain the proliferative nature of leukemic cells. This comprehensive scenario indicates that Tax and HBZ may cooperate for the long-term development and maintenance of leukemic cells in ATL.

### 3.4. Induction of Regulatory T-Cell Differentiation

Similar to regulatory T cells (Tregs), leukemic cells of ATL possess a CD4^+^CD25^+^ phenotype. The forkhead box P3 (FoxP3) is critical for the function of Tregs [5]. FoxP3 expression by HTLV-1 infected T cells is seen in two-thirds of ATL cases [70]. Previous reports indicate that the development and function of Tregs require the TGF-β signaling [71]. Notably, HTLV-1 infected T cells, unlike Tregs, are resistant to growth-inhibitory effect of TGF-β, thus the active TGF-β pathway does not impair the leukemic growth of ATL cells [72,73,74,75]. Recently, we observed that HBZ interacted with Smad2/3, key components of the TGF-β pathway, to form a ternary complex of HBZ/Smad3/p300 that enhanced TGF-β/Smad transcriptional responses in a p300-dependent manner. The enhancement of TGF-β signaling by HBZ results in the overexpression of Foxp3 in naïve T cells [38]. Increased CD4^+^Foxp3^+^ Treg cells were also observed in HBZ-transgenic mice (Figure 2). Furthermore, a luciferase assay validated that HBZ induces transcription of the *Foxp3* gene. Thus, HBZ-induced Foxp3 expression could be a mechanism for the increase of Foxp3^+^ Treg cells *in vitro* and *in vivo* [46].

Numerous viruses have developed strategies to modulate TGF-β signaling using viral proteins. Examples include hepatitis B virus pX; hepatitis C virus core protein, NS3 and NS5; Kaposi sarcoma-associated herpesvirus K-bZIP; and Epstein-Barr virus LMP1 [76,77,78,79]. Like HBZ, the HBV pX and severe acute respiratory syndrome N protein enhance the transcriptional responses of TGF-β. Curiously, these viruses seem to employ a common strategy to nullify the TGF-β signaling by having their viral proteins bind to Smad proteins [76,77,78].

### 3.5. Impaired Cell-Mediated Immunity

Impaired cell-mediated immunity has been demonstrated in HTLV-1 carriers and ATL patients, causing frequent opportunistic infections by various pathogens [80]. However, the mechanism by which HTLV-1 causes immune deficiency has not been well studied. Sugata *et al.* observed that HBZ transgenic mice were highly susceptible to intravaginal infection with herpes simplex virus type 2 (HSV-2) and displayed decreased immune responses to primary and secondary infection with *Listeria monocytogenes* (LM). The production of IFN-γ by CD4^+^ T cells was shown to be suppressed in HBZ-Tg mice [81]. Previous studies have reported that HBZ suppresses host cell signaling pathways that are critical for T-cell immune response, such as the NF-κB, AP-1, and NFAT (Figure 2) [46,54,56,65]. Indeed, HBZ suppresses IFN-γ transcription through interaction with NFAT and c-Jun. Thus, HBZ inhibits cell-mediated immunity *in vivo* by interfering with the host cell signaling pathway, suggesting important roles for HBZ in HTLV-1-induced immunodeficiency.

### 3.6. Induction of T-Cell Lymphoma and Systemic Inflammation

To study the function of HBZ *in vivo*, Satou *et al.* generated transgenic mice expressing HBZ under the control of the mouse CD4 promoter/enhancer, which induces HBZ gene expression specifically in CD4^+^ T cells [46]. Similar to HTLV-1 infected individuals, the majority of HBZ transgenic mice spontaneously developed chronic inflammation in the skin and lungs. Infiltration of CD3^+^CD4^+^ T cells into the dermis and epidermis in the lesions of HBZ-Tg mice was apparent. Moreover, one-third of HBZ-Tg mice developed T-cell lymphomas after a long latent period [46,82]. As observed in HTLV-1-infected individuals, more effector/memory and regulatory CD4^+^ T cells were detected in the HBZ transgenic mice. However, the function of CD4^+^Foxp3^+^ Treg cells in HBZ transgenic mice was impaired, whereas their proliferation increased. As a mechanism, HBZ impairs the suppressive function of Treg cells by binding to Foxp3 and NFAT (Figure 2) [46]. Further studies demonstrated that HBZ-mediated inflammation is closely linked to oncogenesis in CD4^+^ T cells and that IFN-γ is an accelerator of HBZ-induced inflammation [83]. Yamamoto-Taguchi *et al.* reported that iTreg cells increased in HBZ-Tg mice and that Treg cells of HBZ-Tg mice tend to lose Foxp3 expression, leading to increased IFN-γ-expressing proinflammatory cells [84]. Cell adhesion and migration are enhanced in the CD4^+^ T cells of HBZ-Tg mice. Thus, HBZ seems to impair various functions of conventional and regulatory T cells and thereby critically contributes to the development of inflammation *in vivo*.

### 3.7. Differences between HBZ and APH-2

HTLV-1 and HTLV-2 are closely related human retroviruses that have been studied extensively [85,86]. HTLV-1 is associated with ATL and a variety of immune-mediated disorders including HAM/TSP. In contrast, HTLV-2 is much less pathogenic, with only a few cases of variant hairy cell leukemia and neurological disease reported. Similar to HBZ, HTLV-2 also generates an antisense transcript, termed APH-2 (antisense protein of HTLV-2) [87,88]. While most components of HTLV-1 and HTLV-2, including Tax-1 and Tax-2, show high degree of conservation, APH-2 exhibits less than 30% homology to HBZ. Although APH-2 does not harbor a bZIP domain, it can suppress Tax2-mediated viral transcription by interacting with CREB, similar to the effects displayed by HBZ [87]. APH-2 expression was found to correlate with the proviral load in HTLV-2-infected carriers; however, APH-2 did not appear to promote T cell proliferation and lymphocytosis. Arnold *et al.* reported that HBZ is not required for efficient infectivity or HTLV-1-mediated immortalization of primary human T lymphocytes *in vitro* [89]. However, HBZ enhanced infectivity and persistence of HTLV-1 in inoculated rabbits, indicating that HBZ is not required for cellular immortalization but enhances infectivity and persistence *in vivo* [32]. Unlike HBZ, APH-2 is dispensable for enhancing viral replication and persistent infection in the rabbit animal model [90]. In addition, APH-2 could contribute to the lower virulence of HTLV-2 [90]. Thus, the antisense transcripts of HTLV-1 and HTLV-2 exhibit different functions *in vivo*, and further studies are necessary to clarify their distinct pathobiologies.

## 4. Perspectives

It has been 35 years since the discovery of HTLV-1. Numerous studies have focused on elucidating the molecular mechanisms by which HTLV-1-encoded viral proteins induce viral replication, cellular transformation, and oncogenesis. Tax is thought to have an important role in the leukemogenesis of HTLV-1 because of its pleiotropic functions. However, Tax expression is frequently lost during the development of ATL suggesting that Tax may not be essential for this process. By contrast, HBZ is the only viral gene that is constitutively expressed in ATL cases and thus is a plausible player to critically control the ATL leukemogenesis. In addition, APOBEC3G frequently introduces mutations to HTLV-1 genes before proviral integration but HBZ seems to be spared from this alteration. As discussed in this review, current findings support the view that HBZ is indispensable for leukemogenesis by HTLV-1. Further studies are needed to elucidate the precise molecular mechanisms by which HBZ induces oncogenesis so that novel therapies targeting HBZ could be developed.
